# Supplementary material for: Neonatal mortality and associated factors among neonates admitted to neonatal intensive care unit at public hospitals of Somali Regional State, Eastern Ethiopia: A multicenter retrospective analysis
Source: PLoS One. 2022 May 26;17(5):e0268648. doi: 10.1371/journal.pone.0268648 (PMC9135220; doi:10.1371/journal.pone.0268648)
Supplement: Attachment — Submitted filename: 2.Response to reviewers.docx [file pone.0268648.s002.docx]

**Authors’ Response to the editor’s and Reviewers’ comments and Suggestions**

**Manuscript ID:** PONE-D-21-13372

**Journal:** PLOS ONE

**Dear Editors and Reviewers,**

Thank you so much for giving us an opportunity to submit a revised draft of our manuscript entitled “***Neonatal mortality and Associated Factors among Neonates Admitted to Neonatal Intensive Care Unit in Public Hospitals of Somali Regional State, Eastern Ethiopia: A two years’ retrospective Analysis”*** to this high visibility impact factor and peer reviewed Journal. We appreciate the time and effort that you and the reviewers dedicated to providing feedback on our manuscript. We are very grateful for the insightful comments and valuable improvements to our premature paper. We have incorporated most of the **suggestions** and **comments** made by handling editor, reviewers. All comments and suggestions are clearly stated and well addressed (a point-by-point to the reviewer’s comments and concerns). These changes are **highlighted in Red font color** within the clean revised manuscript.

**Authors’ Response to Editor’s Comments and Suggestions**

**Title*: Neonatal mortality and Associated Factors among Neonates Admitted to Neonatal Intensive Care Unit in Public Hospitals of Somali Regional State, Eastern Ethiopia: A two years’ retrospective Analysis***

**Authors:** 1.Hamda Ahmed Mohamed(First author),

2.Zemenu Shiferaw (Co-author),

3.Abdurahman Kedir Roble(Co-author)

4.Mohammed Abdurke Kure(Co-author & Corresponding author)

**To: Handling Editor(s)**

**From: Mohammed Abdurke Kure (Corresponding Author)**

**Subject: Submission of *Incorporated Comments and Suggestions***

First, we thank you for your constructive comments and helpful suggestions that helped us to improve and enrich our manuscript. Here under in the table below, we have pointed out how authors incorporated your valuable comments, suggestions and concerns one by one.

| **Editor’s Comments to the Authors** | **Authors’ Responses to Editor’s Comments and Suggestions** |
| --- | --- |
| **Editor’s General Comments and Suggestions** | **Overall**, thank you so much for **cooperation** to handle our manuscript. Handling paper is really needs dedication and strong commitment. **Thanks a lot**! |
| **1**.Thank you for submitting your manuscript to PLOS ONE. After careful consideration, we feel that it has merit but does not fully meet PLOS ONE’s publication criteria as it currently stands. Therefore, we invite you to submit a revised version of the manuscript that addresses the points raised during the review process. | **Great!** We are very happy and overjoyed. Thank you very much for giving us an opportunity to submit our revised manuscript to such legitimate and high visibility impact factor Journal (PLOS ONE). |
| **2**.Your manuscript was reviewed by 3 experts in the field. The majority identified many important problems in your submission and provided copious comments.  Please revise the manuscript as per the reviewers' comments especially the  results section was difficult to follow. | **Ok, accepted with thanks.** We appreciate the time and effort that you and the reviewers dedicated to providing feedback on our manuscript. |
| **3.**Please consider the reviewers' comments and provide point-by-point responses. | **Thanks a lot.** Dear editor, the authors critically reviewed all comments and suggestions raised during review process and corrected all necessary modifications. The newly modified change were highlighted with red font color in the clean revised main manuscript. |
| **4**.We have noticed that you did not upload your data,  Plos one allows data to be available upon request if there are legal or ethical restrictions on sharing data publicly. | **Ok, great!** No legal restrictions for our data set. We decided to upload all data sets used for analysis in this study without any restrictions. **Thanks a lot!** |
| **Academic Editor’s Specific Comments (Journal Requirements)** |  |
| **1**. Please ensure that your manuscript meets PLOS ONE's style requirements, including those for file naming. The PLOS ONE style templates can be found athttps://journals.plos.org/plosone/s/file?id=wjVg/PLOSOne_formatting_sample_main_body.pdf and | **Thank you so much**. You are perfect**.** These are very important comments. Even it is the authors mandatory to stick to Journal’s format guidelines. Now, the authors addressed this critical issues based on your valuable suggestion. We downloaded all formats templates (PLOS Affiliations Formatting and Manuscript body formatting guidelines) from Journal’s Website, and critically read and corrected all necessary formatting. Newly changed and corrected were highlighted with red font color in the clean revised manuscript. |
| **2**. In the ethics statement in the manuscript and in the online submission form, please provide additional information about the patient records used in your retrospective study | **Thanks.** You are perfect**.** This valid comment was addressed based on your valuable suggestion. Newly changed and corrected was highlighted with red font color in the clean revised manuscript (on page11, Lines=226-232) |
| **3.**Please remove any funding-related text from the manuscript and let us know how you would like to update your Funding Statement. | **Ok, thanks.** The editor is correct. We are very sorry; this is against PLOS ONE authors’ guideline. The authors critically reviewed this valid comment and corrected the necessary modification. The newly modified change was highlighted with red font color in the clean revised manuscript |
| **4.** If there are no restrictions, please upload the minimal anonymized data set necessary to replicate your study findings as either Supporting Information files. We will update your data availability statement on your behalf to reflect the information you provide. | **Thank you very much.** In fact, this is a valid concern. We critically considered this point, and we decided to upload data set used for analysis. |
| **5**. Your ethics statement should only appear in the Methods section of your manuscript. If your ethics statement is written in any section besides the Methods, please delete it from any other section. | **Thank you a lot for such a technical input**. Now, authors considered the raised issue. After thoroughly and critically revised this important comment, we removed from previously it appeared in Acknowledgment part. Thanks! |

**End of authors’ responses for Handling Editor(s)**

**Authors’ Response to Reviewer 1’s Comments and Suggestions**

**Title: Neonatal mortality and Associated Factors among Neonates Admitted to Neonatal Intensive Care Unit in Public Hospitals of Somali Regional State, Eastern Ethiopia: A two years’ retrospective Analysis**

**Authors:** 1.Hamda Ahmed Mohamed(First author),

2.Zemenu Shiferaw (Co-author),

3.Abdurahman Kedir Roble(Co-author)

4.Mohammed Abdurke Kure(Co-author & Corresponding author)

**To: Reviewer 1**

**From:** Mohammed Abdurke Kure (Corresponding Author)

**Subject: Submission of *Incorporated Comments and Suggestions***

First and foremost, we would like to thank you for your constructive and valuable comments and helpful suggestions that helped us to improve and enrich our premature manuscript. Here under in the table we have pointed out how authors incorporated your valuable comments, suggestions and concerns one by one.

| **Reviewer’s Comments to the Authors** | **Authors’ Responses to Reviewer’s comments** |
| --- | --- |
| **Reviewer’s General Comments** | Overall, thank you very much for your positive and constructive suggestions |
| **1**.Is the manuscript technically sound, and do the data support the conclusions?  **Reviewer #1: Yes** | **Thank you very much!!** |
| **2.** Has the statistical analysis been performed appropriately and rigorously?  **Reviewer #1: Yes** | **Thank you so much**. We would like to thank you for your appreciation and constructive suggestion. Now, even we enriched and revised the paper after initial submission. |
| 3. Have the authors made all data underlying the findings in their manuscript fully available?  **Reviewer #1: Yes** | Thanks! |
| 4. Is the manuscript presented in an intelligible fashion and written in standard English?  **Reviewer #1: No** | **Thank you very much** for such valuable intellectual input. The reviewer is correct. Authors critically considered this input. Now, we thoroughly revised and edited the whole parts of our manuscript and extensively corrected all copy-editing errors in the clean revised manuscript. Authors also sent the manuscript to language expert/editor who critically reviewed, edited and corrected all language related errors made in a submitted manuscript. |
| **5. Specific Review’s Comments to the Authors** | **Authors Responses to Reviewers’ Specific comments** |
| **Reviewer #1**: Thank you for the opportunity to read your paper examining predictors and incidence of neonatal mortality in Eastern Ethiopia. | **Thank you very much** for countless effort to review our premature manuscript. |
| I read your paper with interest and think examining these cohorts, especially in the developing world, is important. Generally, I believe the conclusions are fair, albeit limited by the cross-sectional design | **Great! Thanks a lot.** You are perfect. In the cross-sectional study design**,** the researchers cannot draw a conclusion of cause-effect relationship, thanks. |
| My major comment is with respect to “Reviewer Question 4” which relates to clear, correct and unambiguous language, without typographical or grammatical errors. | **Thank you so much**. Authors critically considered this input. Authors acknowledged your efforts. We critically considered and incorporated all raised issues, comments, suggestions and concerns in this manuscript. Moreover, we thoroughly revised and edited the whole parts of our manuscript and extensively corrected all copy-editing errors in the clean revised manuscript. Authors also sent the manuscript to language expert/editor who critically reviewed, edited and corrected all language related errors made in a submitted manuscript. |
| Generally, given multiple errors in syntax & frequent repetition, the manuscript is difficult to read and as such the specifics around methodology are difficult to follow. | **Thank very much.** The reviewer is perfect. At initial submission, the manuscript was not written in concise manner. We critically considered this point. We took a long time, thoroughly and extensively revised and edited the whole manuscript both technically and editorially. The document was also arranged and organized as per journal template. Even we removed all previously written non-sense paragraphs and sentences and replace them with new texts both in ‘**Introduction’** part and **‘Methods’** part. The newly modified change were highlighted with red font color in the clean revised manuscript. |
| There are multiple paragraphs of information that could readily be condensed into 2-3 sentences. I have included some of my feedback below, but this is not all-encompassing as these errors are present throughout the manuscript. | **Thank you a lot**. We appreciated your observation. We considered your deep concern and explicitly incorporated your input both technically and editorially. The document was newly arranged and organized, even we removed all previously written non-sense paragraphs and sentences, clauses and poorly worded text and replaced them with new texts both in ‘**Introduction**’ part and ‘**Methods**’ part. The newly modified change were highlighted with red font color in the clean revised manuscript. |
| **General Comments** |  |
| 1. A punchier title may be considered – perhaps revise to “Neonatal mortality and Associated Factors among Neonates Admitted to the Neonatal Intensive Care Unit in Eastern Ethiopia”. | **Dear, reviewer, thank you very much**. The authors could not understand this suggestion. Do you mean to modify the title of the paper? |
| 2. In tables, precise p values should be stated instead of <0.25 or <0.05 | **Ok! Thank you reviewer.** We really appreciate this valid observation. Is it better to put both p-values and ORs simultaneously**?** But currently, many researchers recommend only ORs instead of using both estimators simultaneously. Anyways, we incorporate this important suggestion based on your recommendation in the tables of bi-variable and multivariable analysis. |
| 3. Figure 2 is likely unnecessary as it represents only a binary pie chart. | **Thank you very much.** In fact, this is a valid concern. We critically considered this important suggestion, and removed previously appeared binary chart, and web substitute it with bar-chart because it is the most appropriate graphical representation for this binary outcome. |
| 4. Generally, I’d suggest removing “died” and replacing with “mortality” where possible. | **Exactly! Thanks.** It is not good to use the word **“died”** to indicate mortality. Based on this important suggestion, we made necessary changes throughout the whole document where possible! |
| **Abstract** | **Thank you a lot**. |
| **INTRODCUTION** | |
| 5. Line 23: Insert “a” between “remains” and “public health challenge” | **Thank you so much.** This was grammatical error. We corrected this on page 2, line 41 of the main manuscript based on your recommendation. |
| 6. Line 25-26: Suggest replace sentence with “In comparison with countries demonstrating the lowest neonatal mortality, the risk of mortality is over 30 times higher in Sub-Saharan Africa”. | **Thank you very much**. The reviewer is correct. We considered this valid concern. Authors carefully considered your valid comments and modified based on your insightful suggestion on page 2, line 24-25 |
| 7. Line 35-36 can be simplified to “identify the effect of predictors on mortality” | **Thank you a lot**. We appreciated your observation. Now authors considered your valid concern and explicitly incorporated your input in the clean revised manuscript and highlighted with red font color |
| 8. Abstract results – suggest include p values | **Thank a lot**. Dear editor, we appreciate your valid suggestion. Is it better to put both p-values and ORs simultaneously in the abstract**?** Most of Biomedical researchers recommend AOR with CIs,(AOR, 95%CI). In previous comment, we incorporate p-value in the tables of bi-variable and multivariable analysis. However, here if add p-value with AOR and 95%CIs, the abstract may become bulk and distorted. Abstract should be short and concise. |
| 9. Line 52-53 in which year? 2017? Consider rewording | **Definitely!** **Thank you so much**. This important comment was modified and highlighted with red font color in the clean revised main manuscript. Thanks. |
| 10. Line 63 – neonatal mortality in India and Pakistan not relevant given not in Africa. Listing rates in these countries is not necessary in this sentence. Also, is this intended to be 38 / 1000 live births, or 38% of neonatal admissions? The sentence reads as all-cause neonatal mortality. | **OK! Thank you**. Here we found 2 important comments. **First.** Neonata Mortality in **Indian** and **Pakistan**: Exactly, you are perfect. We are very sorry! This was unintentionally introduced error during first draft. Actually, the paragraph started with indicating both Africa and Asian regions. However, “Asia” was missed unintentionally. This was editorial error. Now, we corrected this comment **on page 3, line 59-60.** Thank you!  **Second**: NMR should be per 1000 live births. The reviewer is correct. What you are suggesting is the standards. We appreciate this valid observation. Normally, Neonatal mortality can expressed 1000 per live births Rate (NMR=per 1000 live births). However, in **small-scale** research, we can estimate neonatal death in “magnitude” or “proportion” to show the prevalence. Based on your recommendation, we corrected this valid comment, and corrected on page **3, line 61-66.** |
| 11. Line 72 change declined to decline and clarify “but not met national target” as this does not read well & doesn’t make sense here. | **Thank you a lot**. We appreciated your observation. Now authors considered your valid concern and explicitly incorporated your input in the clean revised manuscript and highlighted with red font color |
| 12. Line 83-90 from editorial point of view try to delete some of the “for instance” and “for example” | **Thank you a lot**. We appreciated your observation. Now authors considered your valid concern and explicitly incorporated your input in the clean revised manuscript and highlighted with red font color |
| 13. The introduction is overall a bit wordy and repetitious and could be shortened | **Great! Thank very much.** We have corrected and rephrased based on your recommendation. Even the authors explicitly and critically reviewed this valid comment and rewrote. The previous bulk paragraphs were removed and organized in concise manner. We highlighted the change in red font in the main clean revised manuscript. |
| **Methods** | |
| 14. Need to define “Woredas” and “Kebeles” for international readers. | **Thanks**. Thank you a lot for such technical input. This lacks standards, and difficult to understand by scientific community. We made the correction accordingly. (Woreda=District, Kebeles=The smallest administrative unit in Ethiopia. **Thank you very much!** |
| 15.Line=109–delete“and” | **Great, thanks a lot.** This was editorial error, and we corrected this on page **5, line 106-108** in the clean revised manuscript. |
| 16. Line 114 – “woreda” is not capitalised as previously done. | **Thank you.** The authors have corrected such a language and editing errors. We corrected this grammatical based your insightful comment error here and elsewhere in the clean revised manuscript. |
| 17. Line 122 delete “and” | **Thank you so much.** This was editorial and grammatical error. We corrected this on page 5, lines 126 |
| The methods are particularly difficult to follow given multiple typographical and grammatical errors. I have listed only a few of them above and would suggest a thorough proof reading and rephrasing in many places for clarity. | **Thank you a lot for such a technical input**. You are perfect. Previously, there were bulky paragraphs and sentences in the methods part of this manuscript. Now, the authors considered the raised issue. After thoroughly and critically revised this important comment, we removed all previous bulky texts and long paragraphs/sentences and replaced it with newly corrected one, and all changes were highlighted with red font color in the clean revised manuscript from page 5-9. |
| **Results** |  |
| 18. Line 242 – replace “in” with of” | **Important, Thank you**. We have corrected these unnecessary editing errors based on your valuable recommendations and suggestions. |
| 19. Again multiple typographical errors (including above) which should be proof read and corrected. | **Great! Thanks a lot** for such implicit and critical review for our premature paper. We are very sorry for such unnecessary full of editorial errors for the whole manuscript. |
| **DISCUSSION** | |
| The discussion revolves primarily around comparisons with mortality in other regions and countries. Line 289 – 315 is entirely dedicated to comparator cohorts, of which > 10 are listed. Consider abbreviating this list and condensing this to one paragraph discussing similar cohorts. | **Thank you so much.** We appreciated your observation. The authors critically reviewed this important comment. We condensed and shortened previously bulky long paragraphs based on your valuable suggestions. |
| 20. Line 294 – How is Turkey relevant here? Suggest removing. | **Thank you very much**. In fact, this is a valid concern. We critically considered this point, and modified accordingly. |
| 21. Line 301-302 remove the assertion regarding difference due to time of the study and sample size as this is stated in the following sentence anyway. | **Thanks a lot.** You are perfect**.** This valid comment was incorporated based on your valuable suggestion. Newly changed and corrected was highlighted with Red font color in the Clean revised manuscript. |
| 22. Line 351 delete “Strengths of the study” and “Limitation of the study”. This section needs revising. Standardized+ checklists and questionnaires are not necessarily a strength. | **Thank you again**. You are correct. This is editorial errors and Technical errors. We have corrected this valid concern based on your important implicit suggestion. We delete unnecessary text and highlighted with red font color in the clean revised manuscript. |
| 23. Lines 352-354 is poorly worded. | **Great, Thank you so much**. Very appreciable comment and suggestion. We have corrected these unnecessary poorly worded following your insightful recommendations and suggestions. |
| **Conclusion** |  |
| Conclusions are appropriate but again need to be reworded for simplicity.While I think the results of this study are interesting and relevant to both the scientific community in Ethiopia and the international community, this paper requires major revision, primarily from a language point of view to reduce repetition, simplify the methodology for the reader, and reduce the burden of language errors. | **Thank you very much for such valuable intellectual input**. Authors critically considered this input. Now, we thoroughly revised and edited the whole parts of our manuscript and extensively corrected all copy-editing errors in the clean revised manuscript. Authors also sent the manuscript to language expert/editor who critically reviewed, edited and corrected all language related errors made in a submitted manuscript. |
| **NB: New notification for reviewer 1** | **Dear,** reviewer, thank you very much for your countless effort. Finally, we kindly notify you that during revision of our manuscript, we revised the final model of multivariable analysis and we checked one predictor variable (Birth weight of newborn). The BW was categorized as:  1.LBW= less than 2500mg , 2.Normal BW= 2500-4000mg  3.Macrosomia: >4000mg. In SPSS analysis, initially we used the “**Macrosomia**” as Ref. category, and no significant association was found in multivariable analysis. However, in current revision, the authors critically revise this issue and shifted/substituted the previous Ref. category (>4000mg) to “Normal BW(2500-400mg)”, considering the remaining two categories as exposure. As a result, LBW becomes significant predictor of Neonatal mortality. For further details, we submitted **SPSS data** used for analysis to the Journal as Supplementary File 1(S1). We highlighted newly modified changes with red font color in the Table 3 and 4, and elsewhere in the text. **Thank you very much for your time and consideration.** |

### End of authors responses for Reviewer 1

**Authors’ Response to Reviewer 2’s Comments and Suggestions**

**Title*: Neonatal mortality and Associated Factors among Neonates Admitted to Neonatal Intensive Care Unit in Public Hospitals of Somali Regional State, Eastern Ethiopia: A two years’ retrospective Analysis***

**Authors:** 1.Hamda Ahmed Mohamed(First author),

2.Zemenu Shiferaw (Co-author),

3.Abdurahman Kedir Roble(Co-author)

4.Mohammed Abdurke Kure(Co-author & Corresponding author)

**To: Reviewer 2**

**From:** Mohammed Abdurke Kure (Corresponding Author)

**Subject: Submission of *Incorporated Comments and Suggestions***

First and foremost, we would like to acknowledge you for your constructive and valuable comments and helpful suggestions that helped us to improve and enrich our manuscript. Here under in the table we have pointed out how authors incorporated your valuable comments, suggestions and concerns one by one.

| **Reviewer’s Comments to the Authors** | **Authors’ Responses to Reviewer’s comments** |
| --- | --- |
| **Reviewer’s General Comments** | **Overall,** thank you very much for your positive and constructive suggestions |
| **1**.Is the manuscript technically sound, and do the data support the conclusions?  **Reviewer #1: Yes** | **Thank you very much**! We appreciate your valuable response. |
| **2.** Has the statistical analysis been performed appropriately and rigorously?  **Reviewer #1: Yes** | **Thank you very much.** Authors acknowledged your countless efforts. |
| 3. Have the authors made all data underlying the findings in their manuscript fully available?  **Reviewer #1: Yes** | **Thank you reviewer**! We are very grateful for your response. |
| 4. Is the manuscript presented in an intelligible fashion and written in standard English?  **Reviewer #1: Yes** | **Thanks a lot!** Authors also sent the manuscript to language expert/editor who critically reviewed, edited and corrected all language related errors made in a submitted manuscript. |
| **5. Specific Review’s Comments to the Authors** | **Authors Responses to Reviewers’ Specific comments** |
| **Reviewer #2:** The authors studied predictors of neonatal mortality in 3 public hospitals in the Somali region, Eastern Ethiopia. The research is original; it was the first in this region, which is mainly a rural area. The data presented supported the conclusion and fitted with the study objective. The sample size was calculated appropriately, the sample was randomly selected and the satatistical analysis were rigorously performed. The results were properly discussed and the limitations of the study were mentioned. | **Thank you very much**. We would like to thank you for your appreciation and constructive suggestion. Further, we revised and enriched the paper after previous initial submission to the journal. |
| **Reviewer #2** However, I have some suggestions to improve the manuscripts : | **Thank you a lot**. We accepted all your valid suggestion and concern in this paper, and correct accordingly. |
| 1. There are some grammar and spelling mistakes | **Thank you so much**. Authors critically considered this input. Now, we thoroughly revised and edited the whole parts of our manuscript and extensively corrected all copy-editing errors in the clean revised manuscript. Authors also sent the manuscript to language expert/editor who critically reviewed, edited and corrected all language related errors made in a submitted manuscript. |
| 2. The p-value of the Hosmer-Lemeshow goodness of fitness test is a result and should be included with the table of the final model in the results section with the determination coefficient R2. | **Ok, thanks!** We really appreciate this valid suggestion. Dear reviewer, is it better to put H-L goodness of fitness test result and R2 in Final model of Multivariable analysis? Where do we put this result in table? Do we create its own column? Most of biomedical researchers put the results of H-L goodness test, VIF and tolerance in methods part. Anyways, we incorporate this important suggestion in the clean revised manuscript in the method part. |
| 3. I suggest to present the final model only of the multivariable regression with the goodness of fit criteria. | **Ok! Thank you so much**, reviewer. We also put this for only multivariable logistic regression analysis. |
| **NB: New Notification for Reviewer 2** | **Dear,** reviewer, thank you very much for your countless effort. Finally, we kindly notify you that during revision of our manuscript, we revised the final model of multivariable analysis and we checked one predictor variable (Birth weight of newborn). The BW was categorized as:  1.LBW= less than 2500mg , 2.Normal BW= 2500-4000mg  3.Macrosomia: >4000mg. In SPSS analysis, initially we used the “**Macrosomia**” as Ref. category, and no significant association was found in multivariable analysis. However, in current revision, the authors critically revise this issue and shifted/substituted the previous Ref. category (>4000mg) to “Normal BW(2500-400mg)”, considering the remaining two categories as exposure. As a result, LBW becomes significant predictor of Neonatal mortality. For further details, we submitted **SPSS data** used for analysis to the Journal as Supplementary File 1(S1). We highlighted newly modified changes with red font color in the Table 3 and 4, and elsewhere in the text. **Thank you very much for your time and consideration** |

**End of authors responses to Reviewer 2**

**Authors’ Response to Reviewer 3’s Comments and Suggestions**

**Title*: Neonatal mortality and Associated Factors among Neonates Admitted to Neonatal Intensive Care Unit in Public Hospitals of Somali Regional State, Eastern Ethiopia: A two years’ retrospective Analysis***

**Authors:** 1. Hamda Ahmed Mohamed(First author),

2. Zemenu Shiferaw (Co-author),

3. Abdurahman Kedir Roble(Co-author)

4. Mohammed Abdurke Kure(Co-author & Corresponding author)

**To: Reviewer 3**

**From:** Mohammed Abdurke Kure (Corresponding Author)

**Subject: Submission of *Incorporated Comments and Suggestions***

First and foremost, we would like to thank you for your insightful comments and helpful suggestions that helped us to improve and enrich our manuscript. Here under in the table we have pointed out how authors incorporated your valuable comments, suggestions and concerns one by one.

| **Reviewer’s Comments to the Authors** | **Authors’ Responses to Reviewer’s comments** |
| --- | --- |
| **Reviewer’s General Comments** | Overall, thank you very much for your positive and constructive suggestions |
| **1**. Is the manuscript technically sound, and do the data support the conclusions?  **Reviewer #1: Partly** | Thank you very much. We appreciate this valid constructive suggestion. We critically revised the whole manuscript both technically and editorially to make it more scientifically robust. |
| **2.** Has the statistical analysis been performed appropriately and rigorously?  **Reviewer #1: Yes** | **Thanks** a lot for such positive and constructive suggestion! Further, we frequently revised the statistical analysis, and results to enrich the manuscript than previous its status. |
| **3.** Have the authors made all data underlying the findings in their manuscript fully available?  **Reviewer #1: Yes** | **Thank you so much**. Authors acknowledged for your countless effort. |
| **4**.Is the manuscript presented in an intelligible fashion and written in standard English?  **Reviewer #1: Yes** | **Thank you a lot**. We appreciated your valid observation. Authors also sent the manuscript to language expert/editor who critically reviewed, edited and corrected all language related errors made in a submitted manuscript. |
| **5. Specific Review’s Comments to the Authors** | **Authors Responses to Reviewers’ Specific comments** |
| **Reviewer #3:** The article provides a very nice picture on neonatal mortality and relevant risk factors for neonatal mortality in Ethiopia. It is worthwhile to be published. However, the article is not written carefully, abbreviations are not introduced, and country specific situation are not explained for an international readership and one citation is misleading. | **Great, Thank you so much**. Very appreciable comment and suggestion. Authors acknowledge for your constructive suggestions! |
| **General remarks:** Abbreviation need to be introduced once when used first time, and then this abbreviation shall be used through the whole text. I believe NMR is introduce three times, however ANC, NM, and NICU is not explained at all. Due to the tremendous use of abbreviation, I also suggest providing an abbreviation list where all abbreviations are explained. Please check the correct use of all abbreviation in the text. | **Thanks a lot.** You are perfect. These are very important observation. These valid comments and suggestions were addressed based on your insightful suggestion in the introduction part and elsewhere. All abbreviations/Acronyms (appear at least 3 or more in the document) were critically revised and corrected based on your valuable suggestions. Newly changed and corrected were highlighted with red font color in the clean revised manuscript. |
| **INTRODCUTION** | |
| -The introduction explains the importance of the research question and uses relevant literature. However, the rates from neonatal mortality seems to be wrong, (line 61 ff: information of neonatal mortality is normally given in number per 1000 and not in %; the relevant cited source is connected to under 5 years mortality which does not make sense in this context; and the numbers are not repeatable. Please correct these numbers and cite a correct source. | **Thank you so much.** The reviewer is correct. We considered this valid concern. Authors carefully considered these important comments. We took a long time and extensively reviewed, edited and revised these bolded technical errors. **Here we found 2 important comments.**  **1.NMR**: You are perfect. What you are suggesting is the standards. NMR should be per 1000 live births. The reviewer is correct. What you are suggesting is the standards. We appreciate this valid observation. Normally, Neonatal mortality can expressed 1000 per live births Rate (NMR=per 1000 live births). However, some times, in **small-scale** research, we can estimate these neonatal death in “magnitude” or “proportion” to show the prevalence. Based on your recommendation, we corrected this valid comment, and corrected on page **3, line 61-64.**  **2. Incorrect citation**: **Thanks a lot**. Dear reviewer, we are very sorry! This misused citation was unintentionally introduced during manuscript drafting. Now, we critically considered, and revised our **endnote library** to solve the issues. The newly modified changes were highlighted with red font color in the clean revised main manuscript. |
| -Furthermore, I suggest introducing shortly the Apgar-score as well as ANC and ANC follow up in the introduction. You cannot expect that the international readership knows this words/abbreviations. | **Thank you a lot**. We appreciated your valid observation. Now authors considered your valid concern and explicitly incorporated your input in the **operational definition (in the methods**) **(APGAR Score, ANC and ANC follow up)** and the newly modified changes were highlighted with red font color in the clean revised manuscript. |
| **Methods** | |
| -In the study population sample size, data collection and data analysis are presented carefully. However, the sample size calculation can be presented more condensed. And the coding of neonatal mortality can be dropped from the explanation. | **Thank you a lot for such a technical input**. Now, authors considered the raised issue. After thoroughly and critically revised this important comment, we removed previous bulky and long sentence and replaced with newly corrected one. |
| Furthermore, under analysis it was mentioned that collinearity analysis was done, but the results were not presented. I expect a high collinearity between apgar-score and asphyxia (which is defined via apgar score) and ANC follow up and No of ANC-visits. Therefore, the results are important to present. | **Great,** thanks a lot for such important intellectual input. These are very critical and concerns issues in the final models of multivariable analysis. **Dear, reviewer**, really we appreciate your insightful and logical input. You are perfect. Usually we expect similar predictor variables to have collinearity effect(highly correlation). For example,( primiparity **Vs** multiparity, Apga score **Vs** Birth asphyxia, Grand multipara **Vs** Advance maternal etc..). However, in our case, we didn’t encounter any collinearity effect between “Apgar score” and “Birth asphyxia” in using both VIF and Tolerance. All VIF results were less than 5 and All tolerance results were greater than 0.1. For instance, in our case=  **1.Apgar score (**VIF= 1.44**,** Tolerance=0.70), **2.Ashyxia**(VIF= 1.024, Tolerance=0.97)  3.ANC follow(VIF=1.012, Tolerance=0.98) |
| **Result presentation is nice and carefully done**. | **Thank you so much.** Authors greatly acknowledge for your countless effort. |
| **Table 2:** I suggest to present for binary variables only one line. If you know that 8.6 (n=44) have Hypothermia, then it is implicitly given that the rest do not have hyperthermia this line can be dropped from the table. However, that is relevant only for binary/dichotomous variables. | **Great, Thanks a lot** for such implicit and critical review for our premature paper. Dear reviewer, you are perfect. We really appreciate this valid observation. However, in our case, the questionnaire/tool was prepared in binary response to assess neonatal conditions(Yes or No). Here, our intention is assess presence or absence of hypothermia. Thus. we prepared questions for hypothermia as: Does the neonate have Hypothermia at admission? 1. Yes 2. No, However, there was no question to assess hyperthermia. Thank a lot for your insightful suggestion and recommendation. |
| On the other hand, there is one variable (number of ANC visits) which is not dichotomous/binary, but only presented in this form, clarification is necessary. | **Thanks a lot.** Number of ANC visits were presented in Table 2 as 1. 1-3 visits  2.>3visits….However, we didn’t consider it for regression analysis, instead we selected Presence or absence of ANC follow up. Actually, this is very important concern. |
| **Table 4**: significant results are only marked under AOR but not under COR. Please add this. Furthermore, both abbreviations need to be introduced under the table. | **Thanks a lot.** You are perfect. These are very important observation. These valid comments and suggestions were addressed based on your insightful suggestion in both tables 3 and 4. |
| **Figure 1**. The arrow text line “Simple random sampling (SRS)” can be dropped. The line above contains 196+178+140 which gives exactly 514. No random sampling took place at this stage of sampling procedure. | **Thank you very much.** In fact, this is a valid concern. We critically considered this point, and dropped arrow text line from fig1 based on your valid suggestion. Thank you. |
| **Figure 2:** Can be deleted, The results of this figure can be added in table 2 | **Great! Thanks a lot.** Since it is pie-chat, the binary outcome is not suitable for this figure. Thank you for this intellectual input. Based on your insightful suggestion and your co-reviewers, we modified this figure from previous pie-chart to simple bar-chat. Since it is the major finding of our study, All authors critically discussed on this issue, and decided to change the figure 2 to simple bar-chart for simplicity of the readers(for easily capturing of the magnitude of NM). |
| **DISCUSSION** | |
| The discussion is nicely done, good work. | **Thank you so much.** Authors acknowledged you for your countless effort to review our paper. |
| **NB: New notification for Reviewer 3** | **Dear,** reviewer, thank you very much for your countless effort. Finally, we kindly notify you that during revision of our manuscript, we revised the final model of multivariable analysis and we checked one predictor variable (Birth weight of newborn). The BW was categorized as:  **1.LBW= less than 2500mg , 2.Normal BW= 2500-4000mg**  **3.Macrosomia: >4000mg.** In SPSS analysis, initially we used the “**Macrosomia**” as Ref. category, and no significant association was found in multivariable analysis. However, in current revision, the authors critically revise this issue and shifted/substituted the previous Ref. category (>4000mg) to “Normal BW(2500-400mg)”, considering the remaining two categories as exposure. As a result, LBW becomes significant predictor of Neonatal mortality. For further details, we submitted **SPSS data** used for analysis to the Journal as Supplementary File 1(S1). We highlighted newly modified changes with red font color in the Table 3 and 4, and elsewhere in the text. **Thank you very much for your time and consideration** |

**End of authors’ responses to Reviewer 3**
