# Supplementary material for: Neonatal mortality and associated factors among neonates admitted to neonatal intensive care unit at public hospitals of Somali Regional State, Eastern Ethiopia: A multicenter retrospective analysis
Source: PLoS One. 2022 May 26;17(5):e0268648. doi: 10.1371/journal.pone.0268648 (PMC9135220; doi:10.1371/journal.pone.0268648)
Supplement: Attachment — Submitted filename: 3.Response to Editor= NM.docx [file pone.0268648.s003.docx]

**Authors’ response to the editor’s minor comments and suggestions:**

- **Manuscript ID:** PONE-D-21-13372
- **MS title**: *Neonatal mortality and Associated Factors among Neonates Admitted to Neonatal Intensive Care Unit in Public Hospitals of Somali Regional State, Eastern Ethiopia: A multicenter retrospective Analysis*
- **Authors**: Hamda Ahmed Mohamed^1^, Zemenu Shiferaw^2^, Abdurahman Kedir Roble^3^, Mohammed Abdurke Kure^4^
- **Journal’s name:** PLOS ONE
- **Date**: October 20, 2021

**Dear editor(s), your Excellency!**

First and foremost, we would like to thank PLOS ONE Journal’s editorial office for giving us an opportunity to submit a revised draft of our manuscript entitled “***Neonatal mortality and Associated Factors among Neonates Admitted to Neonatal Intensive Care Unit in Public Hospitals of Somali Regional State, Eastern Ethiopia: A multicenter retrospective Analysis”*** to this high visibility impact factor and peer reviewed Journal. We appreciate the time and effort that you and the reviewers dedicated to providing feedback on our manuscript. We are also very grateful for the insightful comments and suggestions to our paper. We have incorporated minor comments made by handling editor. Newly modified changes were **highlighted in red font color** in the specific part of the revised manuscript.

**Subject: Authors’ Response to Specific Comments and Suggestion of Academic Editor**

**To:** Handling Editor(s)

**From:** Mohammed Abdurke Kure (Corresponding Author)

Above all, authors thank you for your insightful constructive comments and suggestions that helped us to improve and enrich our manuscript to this status. Next, here in the table below, we tried to address your valuable comments, suggestions and concerns one by one.

| **Editor’s Comments and suggestion to the Authors** | **Authors’ Responses to Editor’s Comments and Suggestions** |
| --- | --- |
| **Editor’s General Comments and Suggestions** | **Overall**, thank you so much for **cooperation** to handle our manuscript. Handling paper is really needs dedication and strong commitment. **Thanks a lot**! |
| **1**.Thank you for submitting your manuscript to PLOS ONE. After careful consideration, we feel that it has merit but does not fully meet PLOS ONE’s publication criteria as it currently stands. Therefore, we invite you to submit a revised version of the manuscript that addresses the points raised during the review process. | - **Great!** We are very grateful for the entire editorial office in general, and respective editorial team in particular. - **Further,** above all, we would like to extend our deepest appreciation to the handling academic editor, for the time and countless effort that she/he dedicated to handle our paper as academic editor (handling editor). We understand that handling paper is really needs dedication and strong commitment. - Finally, authors thank you for giving us an opportunity to submit our revised manuscript to such legitimate and high visibility impact factor Journal (PLOS ONE). |
| **2**. Thank you for your response to the reviewer comments. The reviewers consider the manuscript to be much improved. However, some additional editorial concerns must be addressed before the manuscript can be considered for publication. | - **Ok, we accepted with thanks.** We appreciate the time and effort that you and the reviewers have dedicated to providing feedback on our manuscript. - We critically revised all editor’s concerns and comments, and we addressed all accordingly(on respective page numbers). |
| **3.** Specifically, we found a degree of text overlap between your submission and the following previously published works:  **a.** In lines 55-60, there is some overlap with http://www.kma.org.kw/uploads/versions/EEQGAXUUOAQIGLDPHVORFANO.pdf (pages113-114)  **b.** In lines 81-85 there is some overlap with https://documents1.worldbank.org/curated/en/384721537219780286/pdf/129971-AR-PUBLIC-UN-IGME-Child-Mortality-Report-2018.pdf?fbclid=IwAR2X1_3wyyZFl_v1-6yrBvZgIM0jMjnw4cJL4JxRBou8XV_eJfo2oGOyg20 (page 12) | - **Thanks a lot.** Really, this is very critical concerns. Dear editor, we are very sorry for such unnecessary bolded mistake made in the previous last submission. In this regard, we observed two important suggestion:   **a. overlap issues of lines 55-60:**   - Dear editor, these overlapping/similarity texts were **unintentionally** introduced during the 2^nd^ round of revised submission. It’s all our fault and therefore, we sincerely **apologize** for all these an unintentionally introduced technical errors. - These valid comments were extensively revised, and addressed based on your implicit and valuable suggestion. Newly changed and corrected parts were highlighted with red font color in the clean revised manuscript (please see page=3, lines: 54-64). **Thank you once again**.   **b. overlap issues of lines 81-85:**   - **Thank you so much:** The editor is perfect. - Similarly, this valid observation was also critically revised and rephrased based on your insightful suggestions. The newly modified change were highlighted with a red-font color in the clean revised manuscript.(Please see, page 4 & 5, lines: 83-97). |
| **4**. Please review the entire manuscript, especially the abovementioned sections to ensure that you rephrase any duplicated text and cite your sources in full. In addition, please review the manuscript again to check for any remaining typographical and grammatical errors, particularly in the Abstract. | - **Thank you very much**. In fact, this is very important suggestion. We are very grateful for this implicit comment and suggestion. Thank you so much for such a valuable intellectual input. Authors critically considered this valid input. - Accordingly, we thoroughly revised and edited not only the abstract, but also the whole parts of our manuscript. - In addition, we extensively corrected all copy-editing errors made in the previous submission. Authors also sent the manuscript to language expert/editor who critically reviewed, edited and corrected all language related errors made in a submitted manuscript. - Finally, the newly modified change were highlighted with red font color in the clean revised manuscript (Page 2). |
| **5**. Please review your reference list to ensure that it is complete and correct. If you have cited papers that have been retracted, please include the rationale for doing so in the manuscript text, or remove these references and replace them with relevant current references. Any changes to the reference list should be mentioned in the rebuttal letter that accompanies your revised manuscript. If you need to cite a retracted article, indicate the article’s retracted status in the References list and also include a citation and full reference for the retraction notice. | **Thank you so much.** You are perfect. The issues of reference are very critical, and have to be revised and corrected before any decision for the acceptance of the Manuscript for publication.   - Even it is the authors mandatory to critically review the references styles and its relevancy (Both in text-citation and Bibliography) to avoid any inconsistence and wrong citations across the document. - We took a long time, thoroughly and critically revised our **endnote library**. Moreover, any wrong citations were checked in the word document and refined accordingly. - Further, in the document citation, we checked the references for any repetition, incompleteness, and inconsistences. - Finally, we addressed these important concerns based on your insightful suggestions. Newly corrected references were highlighted with red font color in the clean revised manuscript.   **In summary**:  Overall, thank you all for your unquantified effort to enrich this manuscript by forwarding your insightful intellectual input. We are very grateful for all reviewers and the entire editorial office in general and respective academic editor in particular. Dear editor, we learned a lot from all steps of review process made in this manuscript. Really, publications process is learning forum.  **=All we can is thanks!!** |

**End of authors’ responses for Handling Editor(s)**
